# Evaluation of gradient strip diffusion for susceptibility testing of aztreonam–avibactam in metallo-β-lactamase-producing Enterobacterales

**DOI:** 10.1128/jcm.00649-24

**Published:** 2024-09-30

**Authors:** Jamie K. Lemon, Cheryl Jankowsi-Romano, Scott Duong, Stefan Juretschko, Vincent A. Streva

**Affiliations:** 1 Northwell Health Clinical Laboratories, New York, New York, USA; 2 Donald and Barbara Zucker School of Medicine at Hofstra/Northwell, Uniondale, New York, USA; Johns Hopkins University, Baltimore, Maryland, USA

**Keywords:** metallo-beta-lactamase, aztreonam–avibactam, antimicrobial susceptibility testing, NDM, gradient strip diffusion

## Abstract

The emergence of metallo-β-lactamase (MBL)-producing Enterobacterales presents unique clinical treatment challenges. Recently developed β-lactam/ β-lactamase inhibitor combination agents, while effective against other carbapenemase-producing organisms, are notably ineffective against MBL producers. While MBLs do not hydrolyze monobactams (aztreonam), many MBL-producing organisms are resistant to aztreonam through alternate mechanisms, leaving cefiderocol as the sole monotherapy treatment option recommended for MBL producers. Recent guidelines for the treatment of MBL-harboring organisms have added combination therapy with aztreonam and ceftazidime–avibactam, using ceftazidime–avibactam as a source of the β-lactamase inhibitor avibactam. Current laboratory testing options for the combination of aztreonam–avibactam are limited to broth microdilution (BMD) and broth disk elution (BDE) methods, which are not practical in most clinical laboratories. In this study, we evaluated the performance of aztreonam/avibactam gradient strips on 103 MBL-producing Enterobacterales patient isolates as well as an additional 31 isolates from the CDC AR Bank. All MBL Enterobacterales patient isolates included in this study harbored a New Delhi metallo-β-lactamase (*bla*_NDM_) gene. Essential agreement of gradient strip minimal inhibitory concentrations (MICs) for patient isolates compared to BMD was 93.2%. While there are no established breakpoints for aztreonam–avibactam, category agreement (CA) for patient isolates was 97.1% when using the CLSI aztreonam breakpoints. There were no major or very major errors observed. There were three minor errors. Precision for aztreonam–avibactam gradient strip diffusion was 100%. These data demonstrate that the use of gradient strip diffusion for aztreonam–avibactam MIC determination in MBL-producing Enterobacterales is a viable option for clinical laboratories.

## INTRODUCTION

The first description in 2009 of *bla*_NDM_-positive, carbapenem-resistant *Klebsiella pneumoniae* was reported in a patient with urinary tract infection, who recently returned from travel to New Delhi, India (1). While initially geographically restricted to the Indian subcontinent, *bla*_NDM_ has spread worldwide, with infections reported in all continents (1–5). While primarily implicated in urinary tract infections, *bla*_NDM_-harboring organisms are increasingly isolated outside the urinary tract (6–9). *bla*_NDM_ is the most prevalent member of the metallo-β-lactamase (MBL) family, which are a class of carbapenem-hydrolyzing enzymes requiring metal ions for their function (10). Infections caused by MBL-producing organisms frequently have poor clinical outcomes and are responsible for disproportionate morbidity and mortality (11).

Isolates harboring *bla*_NDM_ are notable, in that they are resistant to all β-lactam antibiotics, with the exception of aztreonam (1). While *bla*_NDM_ does not hydrolyze aztreonam, organisms harboring *bla*_NDM_ genes often express additional β-lactamases, rendering aztreonam an ineffective therapeutic option. The Infectious Diseases Society of America (IDSA) practice guidance-recommend treatment of non-urinary tract infections caused by Enterobacterales harboring *bla*_NDM_ with either cefiderocol monotherapy or ceftazidime–avibactam in combination with aztreonam. This combination therapy utilizes avibactam as an inhibitor of the other β-lactamases present in *bla*_NDM_-positive isolates, allowing aztreonam to regain efficacy against these organisms (12). Although ceftazidime–avibactam plus aztreonam combination therapy is beginning to be used clinically for the treatment of infections caused by *bla*_NDM_-harboring organisms, there is no FDA-approved combination drug, and correspondingly, no FDA-cleared laboratory tests for antimicrobial susceptibility and no interpretive guidelines for AST testing.

The Clinical and Laboratory Standards Institute (CLSI) recognizes two testing options for the evaluation of the *in vitro* activity of aztreonam–avibactam: 1) broth microdilution (BMD) and 2) broth disk elution (BDE), which is tested using aztreonam plus ceftazidime–avibactam disks (13, 14). Both methods represent specialized testing that may not be widely utilized in the majority of clinical laboratory settings, where commercial AST platforms predominate.

In this study, we evaluated a research using only the gradient strip diffusion test for the determination of minimal inhibitory concentrations (MIC) of aztreonam–avibactam in Enterobacterales isolates producing a metallo-β-lactamase.

## MATERIALS AND METHODS

### Bacterial isolates

Isolates from routine clinical specimens (*n* = 103) submitted to the Northwell Health Microbiology Laboratory between May and November 2023 were selected for inclusion in the study based on identification of a member of the Enterobacterales demonstrating phenotypic resistance to at least a single carbapenem agent and detection of a metallo-β-lactamase by either multiplex blood culture identification PCR (BCID2, BioMerieux Inc., Salt Lake City, UT) or lateral flow immunoassay (NG-Test CARBA 5, Hardy Diagnostics, Santa Maria, CA). Organism identification was performed by MALDI-ToF-MS (Bruker Daltonics, Billerica, MA). The clinical isolates in this study were obtained from unique patients and included *Klebsiella pneumoniae* (*n* = 76), *Escherichia coli* (*n* = 17), *Enterobacter cloacae* complex (*n* = 6), *Klebsiella oxytoca* (*n* = 2), and *Klebsiella variicola* (*n* = 2) from various specimen types, as described in Table 1. Selected organisms from the following Centers for Disease Control and Prevention (CDC) and Food and Drug Administration (FDA) AR Bank panels were included in this study: Enterobacterales Carbapenem Breakpoint (BIT), Gram-Negative Carbapenemase Detection (CarbaNP), Enterobacterales Carbapenemase Diversity (CRE), and Multi-Mechanism (MULTI). AR Bank specimens for which aztreonam–avibactam MIC data were known (*n* = 31) were acquired from the CDC as frozen glycerol stocks, which were subcultured to 5% Sheep’s Blood Agar (SBA) plates and incubated overnight at 37°C. Isolates were then passaged to SBA one time prior to susceptibility testing. A list of CDC AR Bank isolates included in the study is found in Table S1.

**TABLE 1:** Specimen sources and organisms for clinical isolates^*a*^

Organism	Sources							
	Urine	Blood	Respiratory	Wound	Tissue	Abscess	Joint	Total
*Klebsiella pneumoniae*	37	15	8	10	3	2	1	76 (73.8%)
*Escherichia coli*	12	4	1	-	-	-	-	17 (16.5%)
*Enterobacter cloacae* complex	4	1	-	-	-	1	-	6 (5.8%)
*Klebsiella oxytoca*	-	-	2	-	-	-	-	2 (1.9%)
*Klebsiella variicola*	2	-	-	-	-	-	-	2 (1.9%)
TOTAL	55 (53.4%)	20 (19.4%)	11 (10.7%)	10 (9.7%)	3 (2.9%)	3 (2.9%)	1 (1.0%)	103 (100%)

^*a*^ The number and percentage of clinical isolates by specimen type and organism tested in the study are shown. and majority of isolates were from urine (55/103, 53.4%). Isolates from blood (20/103, 19.4%), respiratory sources (11/103, 10.7%), and wounds (10/103, 9.7%) comprised other major sources in the study sample. Tissues, abscesses, and joint sources (7/103, 6.8%) comprised a minority of sources. Majority of isolates were *Klebsiella pneumoniae* (76/103, 73.8%), followed by *Escherichia coli* (17/103, 16.5%). Other species of the Enterobacterales, including *Enterobacter cloacae* complex, *Klebsiella oxytoca*, and *Klebsiella variicola,* made up a minority of isolates in the study (10/103, 9.7%). Sources for which no organism were recovered are indicated with a dash.

### Aztreonam–avibactam susceptibility testing

Aztreonam–avibactam gradient strip diffusion testing (Liofilchem Inc., Waltham MA) was performed as per the manufacturer’s instructions by preparing a 0.5 McFarland suspension of each bacterial isolate and streaking a lawn onto a Mueller–Hinton Agar (MHA) plate (Becton Dickinson, Sparks, MD), followed by placing a gradient strip. Plates were incubated at 37°C for 16–20 hours prior to reading. MIC values were determined as the value where zones of complete growth inhibition intersect the gradient strip. MICs between two doubling dilutions were rounded up to the higher MIC value. Broth microdilution (BMD) testing for both aztreonam and aztreonam–avibactam was performed by the New York State Department of Health Wadsworth Center using standard methods (15).

### Precision/reproducibility

Precision testing was performed using three ATCC reference strains (*Escherichia coli* ATCC 35218, *Pseudomonas aeruginosa* ATCC 27853, and *Klebsiella pneumoniae* ATCC 700603) and two CDC/FDA AR Bank Isolates (*K. pneumoniae* CDC AR 146 and *E. coli* CDC AR 137). For intra-day precision, 10–20 replicates of each organism were tested in parallel on a single day of testing. For inter-day precision, 45–107 replicates were tested across multiple days. Modal MIC was determined by calculating the mode of all replicates tested for each organism per analysis period.

### Determination of resistance gene subtypes

Clinical isolates were submitted to the CDC Expanded Antimicrobial Susceptibility Testing for Hard-to-Treat Infections (ExAST) program at NYS Department of Health Wadsworth Center for whole-genome sequencing determination of genetic markers of carbapenem resistance, including carbapenemase subtyping.

### Data analysis

Minimal inhibitory concentration (MIC) results from both gradient strip testing and BMD were collated. Essential agreement, category agreement, very major error rate, major error rate, and minor error rate were calculated according to Clinical and Laboratory Standards Institute (CLSI) guidelines (16). Because there are currently no CLSI nor FDA breakpoints for aztreonam–avibactam, the FDA-recognized CLSI aztreonam breakpoints were used to interpret the results.

## RESULTS

### Characterization of clinical isolates

Clinical isolates of metallo-β-lactamase-producing Enterobacterales recovered in our clinical laboratory between May and November 2023 were selected for aztreonam–avibactam susceptibility testing by both broth microdilution (BMD) and gradient strip diffusion (GSD). A total of 103 clinical isolates were tested and represented seven specimen sources (Table 1). The majority of isolates were recovered in urine culture (55/103, 53.4%), followed by blood culture (20/103, 19.4%), respiratory culture (11/103, 10.7%), wound culture (10/103, 9.7%), tissue culture (3/103, 2.9%), abscess culture (3/103, 2.9%), and joint culture (1/103, 1%). The clinical isolates were predominantly *Klebsiella pneumoniae* (76/103, 73.8%), followed by *Escherichia coli* (17/103, 16.5%), *Enterobacter cloacae* complex (6/103, 5.8%), *Klebsiella oxytoca* (2/103, 1.9%), and *Klebsiella variicola* (2/103, 1.9%). All 103 Enterobacterales isolates included in this study harbored a New Delhi metallo-β-lactamase (*bla*_NDM_). The most commonly identified *bla*_NDM_ gene was *bla*_NDM-1_, followed by *bla*_NDM-5_. Six isolates (5.8%) were positive for multiple carbapenemases, each harboring a second carbapenemase gene in addition to *bla*_NDM_. Of these, three isolates also harbored *bla*_KPC_ genes and three isolates also harbored *bla*_OXA-48-like_ genes. The predominant carbapenemase gene harbored by *Klebsiella* species was *bla*_NDM-1_, whereas *bla*_NDM-5_ was more frequently observed in the other Enterobacterales. All isolates tested were not susceptible to aztreonam by the FDA-recognized CLSI interpretive criteria (13).

### Evaluation of aztreonam–avibactam gradient strips on patient isolates

**Fig 1:**
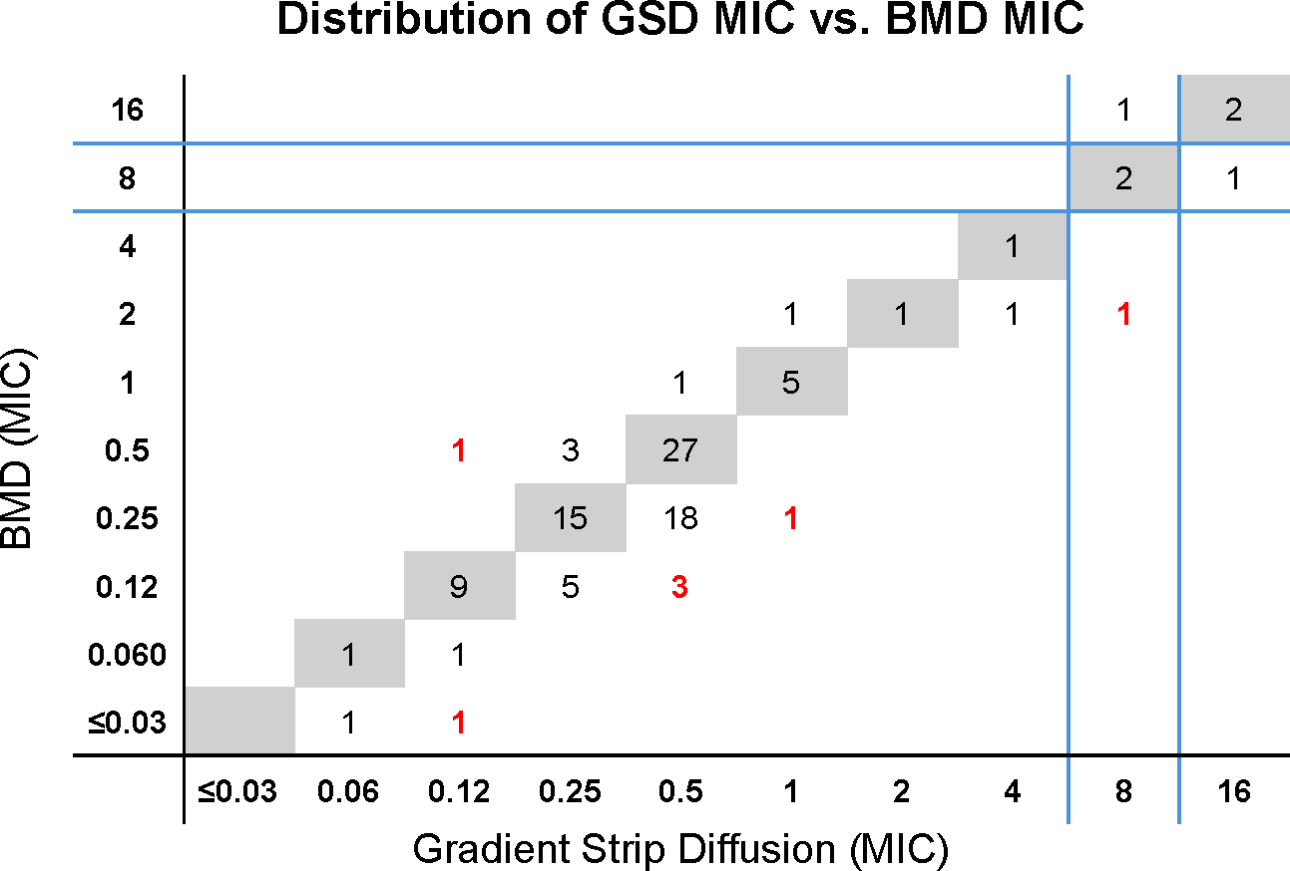
Distribution of aztreonam–avibactam gradient strip diffusion MIC compared to broth microdilution for clinical isolates. The distribution of aztreonam–avibactam MIC as tested by gradient strip diffusion (GSD) compared to the MIC measured by the comparator method (broth microdilution, BMD) for the clinical (*n* = 103) isolates included in this study is shown. Isolates with perfect MIC correlation are shaded in gray, and majority of isolates (63/103, 61.2%) were in this category. Isolates with a GSD MIC that measured two or greater doubling-dilutions different from the comparator BMD MIC are highlighted in red, and a minority of isolates (7/103, 6.8%) were in this category. There are currently no published interpretive breakpoints for aztreonam–avibactam. Blue lines represent the Clinical and Laboratory Standards Institute (CLSI) breakpoints for aztreonam as a surrogate for proposed aztreonam–avibactam breakpoints. Using these surrogate breakpoints, there were three isolates that would have incorrect interpretations by GSD. All three would be categorized as minor errors.

Minimal inhibitory concentration (MIC) values for gradient strip diffusion demonstrated an overall essential agreement (EA) of 93.2% (Table 2). The essential agreement was greater than 90% for all organisms tested, with the exception of *E. cloacae* complex, which exhibited an EA of 83.3%. Of the seven Enterobacterales isolates not in essential agreement, 100% of the gradient strip results were within two doubling-dilutions of the comparator BMD MIC (Fig. 1).

**TABLE 2:** Antimicrobial susceptibility testing results by organism^*a*^

Organism	BMD AZT MIC distribution			BMD AZT/AVI MIC distribution			Gradient strip AZT/AVI MIC distribution			Essential agreement with BMD	Category agreement with BMD
	≤4 (S)	8 (I)	≥16 (R)	≤4/4 (S)	8/4 (I)	≥16/4 (R)	≤4/4 (S)	8/4 (I)	≥16/4 (R)		
Clinical isolates											
*Klebsiella pneumoniae* (*n* = 76)	0 (0%)	0 (0%)	76 (100%)	76 (100%)	0 (0%)	0 (0%)	76 (100%)	0 (0%)	0 (0%)	71/76 (93.4%)	76/76 (100%)
*Escherichia coli* (*n* = 17)	0 (0%)	1 (5.9%)	16 (94.1%)	11 (64.7%)	3 (17.6%)	3 (17.6%)	10 (58.8%)	4 (23.5%)	3 (17.6%)	16/17 (94.1%)	14/17 (82.4%)
*Enterobacter cloacae* complex (*n* = 6)	0 (0%)	0 (0%)	6 (100%)	6 (100%)	0 (0%)	0 (0%)	6 (100%)	0 (0%)	0 (0%)	5/6 (83.3%)	6/6 (100%)
*Klebsiella oxytoca* (*n* = 2)	0 (0%)	0 (0%)	2 (100%)	2 (100%)	0 (0%)	0 (0%)	2 (100%)	0 (0%)	0 (0%)	2/2 (100%)	2/2 (100%)
*Klebsiella variicola* (*n* = 2)	0 (0%)	0 (0%)	2 (100%)	2 (100%)	0 (0%)	0 (0%)	2 (100%)	0 (0%)	0 (0%)	2/2 (100%)	2/2 (100%)
TOTAL (*n* = 103)	0 (0%)	1 (1.0%)	102 (99.0%)	97 (94.2%)	3 (2.9%)	3 (2.9%)	96 (93.2%)	4 (3.9%)	3 (2.9%)	96/103 (93.2%)	100/103 (97.1%)
AR bank isolates											
*Klebsiella pneumoniae* (*n* = 12)	2 (16.7%)	0 (0%)	10 (83.3%)	12 (100%)	0 (0%)	0 (0%)	12 (100%)	0 (0%)	0 (0%)	12/12 (100%)	12/12 (100%)
*Escherichia coli* (*n* = 8)	1 (12.5%)	1 (12.5%)	6 (75.0%)	6 (75.0%)	1 (12.5%)	1 (12.5%)	4 (50.0%)	3 (37.5%)	1 (12.5%)	8/8 (100%)	6/8 (75.0%)
*Enterobacter cloacae* complex (*n* = 6)	0 (0%)	0 (0%)	6 (100%)	6 (100%)	0 (0%)	0 (0%)	6 (100%)	0 (0%)	0 (0%)	5/6 (83.3%)	6/6 (100%)
*Klebsiella aerogenes* (*n* = 1)	0 (0%)	0 (0%)	2 (100%)	2 (100%)	0 (0%)	0 (0%)	2 (100%)	0 (0%)	0 (0%)	2/2 (100%)	2/2 (100%)
*Citrobacter freundii* (*n* = 1)	1 (100%)	0 (0%)	0 (0%)	1 (100%)	0 (0%)	0 (0%)	1 (100%)	0 (0%)	0 (0%)	1/1 (100%)	1/1 (100%)
*Klebsiella oxytoca* (*n* = 1)	1 (100%)	0 (0%)	0 (0%)	1 (100%)	0 (0%)	0 (0%)	1 (100%)	0 (0%)	0 (0%)	1/1 (100%)	1/1 (100%)
*Serratia marcescens* (*n* = 1)	1 (100%)	0 (0%)	0 (0%)	1 (100%)	0 (0%)	0 (0%)	1 (100%)	0 (0%)	0 (0%)	1/1 (100%)	1/1 (100%)
TOTAL (*n* = 31)	6 (19.4%)	1 (3.2%)	24 (77.4%)	29 (93.5%)	1 (3.2%)	1 (3.2%)	27 (87.1%)	3 (9.7%)	1 (3.2%)	30/31 (96.8%)	29/31 (93.5%)
GRAND TOTAL (*n* = 134)	6/134 (4.5%)	2/134 (1.5%)	126/134 (94.0%)	126/134 (94.0%)	4/134 (3.0%)	4/134 (3.0%)	123/134 (91.8%)	7/134 (5.2%)	4/134 (3.0%)	126/134 (94.0%)	132/134 (98.5%)

^*a*^ Susceptibility results for aztreonam (AZT) by broth microdilution (BMD), aztreonam–avibactam (AZT/AVI) by BMD, and AZT/AVI by gradient strip diffusion (GSD) are shown. For AZT, CLSI breakpoints are used to categorize the susceptibility phenotype. For AZT/AVI, the CLSI breakpoints for AZT are used. The essential agreement (EA) and category agreement (CA) for the GSD method compared to the comparator BMD method by organism tested are shown. EA was defined as an MIC result within one doubling-dilution of the MIC value from the comparator method, and CA was defined as agreement of interpretation (susceptible, intermediate, or resistant) with the comparator method. GSD demonstrated high overall essential agreement in both clinical isolates and CDC AR bank isolates (94.0%). The lowest EA was observed for *Enterobacter cloacae* complex (83.3%). Overall category agreement for the GSD method was also high (98.5%). Of note, all isolates that did not demonstrate category agreement were *Escherichia coli*, resulting in a significantly lower CA for this organism (80.0%).

As there are no established breakpoints for aztreonam–avibactam, category agreement was calculated using the CLSI/FDA breakpoints for aztreonam. Using these breakpoints, 97/103 (94.2%) isolates were susceptible to aztreonam–avibactam by BMD, 3/103 (2.9%) isolates had intermediate resistance, and 3/103 (2.9%) isolates were resistant. Overall category agreement was 97.1%. Category agreement was 100% for all organisms tested with the exception of *E. coli*, which displayed a category agreement of 82.4% (Table 2).

All the three isolates not in category agreement resulted in minor errors. One isolate was susceptible by BMD (MIC = 2/4) and showed intermediate resistance by gradient strip (MIC = 8/4). One isolate showed intermediate resistance by BMD (MIC = 8/4) and was shown to be resistant by gradient strip (MIC = 16/4). The final isolate was resistant by BMD (MIC = 16/4) and showed intermediate resistance by gradient strip (MIC = 8/4). Only the first of these three isolates was not in essential agreement.

### Evaluation of aztreonam–avibactam gradient strips on CDC AR bank isolates

CDC AR Bank Enterobacterales isolates (*n* = 31) with well-characterized BMD susceptibility results and defined β-lactamase gene identity were tested using aztreonam–avibactam gradient strips. The AR Bank isolates were predominantly *K. pneumoniae* (38.7%), followed by *E. coli* (25.8%), *E. cloacae* complex (19.4%), *K. aerogenes* (6.5%), and other species of the Enterobacterales, which each consisted of only a single isolate (Table 2). Of the 31 isolates, 25 (80.6%) isolates were not susceptible to aztreonam, while 29 (93.5%) were susceptible to aztreonam–avibactam by gold-standard BMD. The majority (54.8%) of isolates harbored a single carbapenem resistance gene, while 41.9% harbored multiple carbapenemases. One isolate (3.2%) was not sequenced to determine the genetic markers of carbapenem resistance. One isolate showed intermediate resistance to aztreonam–avibactam, and one isolate was resistant. MIC values for gradient strip diffusion exhibited an essential agreement of 96.8%. The single isolate not in essential agreement was an *Enterobacter cloacae* complex that exhibited a two doubling-dilution difference between BMD and gradient strip methods; however, this isolate was interpreted as susceptible using both methods and was therefore in category agreement. Category agreement was 93.5%, with two isolates susceptible by BMD and intermediate by gradient strip diffusion. These two isolates were within a single doubling-dilution of the BMD MIC result. There were no very major or major errors observed. The minor error rate was 2/31 (6.5%). Combined performance results for both clinical isolates and CDC AR Bank isolates are presented in Fig. S1.

### Aztreonam–avibactam gradient strip precision

Precision and reproducibility testing was performed using three quality control strains from the American Type Culture Collection (ATCC, Manassas, VA). Each of the three quality control strains was tested in multiple replicates on a single day to determine the intra-day precision. Additionally, isolates were tested daily in single replicates for multiple days to determine the inter-day precision. For all three control isolates, 100% of the replicates tested were within one doubling-dilution of the modal MIC, and all results were within the expected QC ranges (Table 3).

**TABLE 3:** Aztreonam–avibactam gradient strip precision testing results^*a*^

QC strain/ reference isolate	Expected MIC range (µg/mL)	Intra-day precision				Inter-day precision			
		Observed MIC range	Modal MIC (µg/mL)	Replicates at modal MIC (µg/mL)	Replicates ±1 DD of modal MIC (µg/mL)	Observed MIC range	Modal MIC (µg/mL)	Replicates at modal MIC (µg/mL)	Replicates ±1 DD of modal MIC (µg/mL)
*E. coli* ATCC 35218	0.016/4– 0.06/4	0.016/4–0.031/4	0.031/4	9/10 (90.0%)	10/10 (100%)	0.016/4– 0.06/4	0.031/4	77/107 (72.0%)	107/107 (100%)
*P. aeruginosa* ATCC 27853	2/4–8/4	4/4	4/4	10/10 (100%)	10/10 (100%)	2/4–8/4	4/4	80/107 (74.8%)	107/107 (100%)
*K. pneumoniae* ATCC 700603	0.06/4– 0.5/4	0.25/4	0.25/4	20/20 (100%)	20/20 (100%)	0.25/4	0.25/4	45/45 (100%)	45/45 (100%)
*K. pneumoniae* CDC AR 146	0.25/4	0.125/4– 0.25/4	0.25/4	18/20 (95%)	20/20 (100%)	0.125/4– 0.25/4	0.25/4	43/45 (96%)	45/45 (100%)
*E. coli* CDC AR 137	16/4	8/4–16/4	8/4	14/20 (70%)	20/20 (100%)	8/4–16/4	8/4	35/45 (78%)	45/45 (100%)

^*a*^ Results of precision testing for aztreonam–avibactam gradient strip diffusion (GSD) of two ATCC quality control strains are shown. Both intra-day and inter-day precision were assessed. For each isolate, the modal MIC was within the expected MIC range for the QC organism. All precision replicates for both QC strains were within one doubling-dilution (DD) of the modal MIC.

## DISCUSSION

While the presence of metallo-β-lactamase-harboring isolates of Enterobacterales has been widely described globally, surveillance studies of carbapenem-resistant organisms in North America and Europe have shown that serine β-lactamases (e.g., *bla*_KPC_) have predominated (2). Given that our study identified more than 100 patients harboring MBL Enterobacterales isolates in a 6-month period in our large multi-hospital healthcare system, the landscape of carbapenem resistance genes may be evolving; however, additional surveillance studies are necessary to fully characterize the scope of these potential shifts. In environments where patients can harbor either serine or metallo-β-lactamases, distinguishing the class of β-lactamase responsible for an infection in a clinical setting is critical to ensure appropriate antimicrobial therapy.

While infections caused by serine β-lactamase (e.g., *bla*_KPC_ and *bla*_OXA_)-producing organisms can generally be treated with β-lactam combination agents (e.g., meropenem–vaborbactam, ceftazidime–avibactam, and imipenem–relebactam), these agents are ineffective in the treatment of MBL-producing organisms (17). Treatment of MBL-harboring organisms is difficult and generally involves either monotherapy with cefiderocol or combination therapy with aztreonam and ceftazidime–avibactam (aztreonam–avibactam) (17, 18).

Because knowledge of the mechanism of carbapenem resistance drives treatment choices, clinical laboratories should have the ability to identify and classify the major carbapenemases from routine clinical isolates. Additionally, laboratories should be equipped to provide antimicrobial susceptibility testing for antimicrobial agents required to treat these infections. Cefiderocol antimicrobial susceptibility testing (AST) is available by both broth dilution and disk diffusion. While broth dilution testing requires the use of iron-depleted, cation-adjusted Mueller–Hinton broth, disk diffusion testing can be performed on routine Mueller–Hinton agar. Antimicrobial susceptibility testing for aztreonam–avibactam is more limited and is predominantly performed by broth microdilution, which is not widely available in most clinical settings. Recently, AST testing for aztreonam–avibactam has also been described using broth disk elution; however, this testing methodology has yet to be widely adopted by clinical laboratories (14).

In this study, we evaluated the performance of gradient strip diffusion as a method for determination of MIC values for aztreonam–avibactam in metallo-β-lactamase-producing isolates of Enterobacterales. Gradient strip diffusion is a commonly used methodology in clinical laboratories for the determination of MIC values for a broad range of antimicrobial/organism combinations and can be performed on standard culture media widely available in most clinical laboratories. Overall, we observed high essential agreement of gradient strips when compared to broth microdilution, and when aztreonam breakpoints are used, a similarly high category agreement was observed. For both clinical and CDC AR bank isolates, the lowest essential agreement was seen in isolates of the *Enterobacter cloacae* complex (83.3%). However, the number of *E. cloacae* isolates included in the study is low (*n* = 12), and both isolates not in essential agreement were within two doubling-dilutions of the comparator method. While overall assay agreement was high, the MICs observed by GSD appear to skew one doubling-dilution higher than BMD (35/134 isolates) compared to one doubling-dilution lower than BMD (11/134 isolates). The reason for this difference is undetermined. A notable exception to the strong performance of GSD was the relatively worse performance observed in clinical isolates of *E. coli*. Of note, all six clinical isolates with aztreonam–avibactam MIC values greater than 4/4 µg/mL were *E. coli*. Interestingly, of the 14 *E. coli* isolates for which *bla*_NDM_ genetic information is available, all contained *bla*_NDM-5_. This observation is consistent with descriptions of a high-risk *bla*_NDM-5_ positive sequence type 167 (ST 167) *E. coli* clone described globally from human clinical infections (19–23). Whether the *E. coli* isolates in our study represent ST 167 was not interrogated as part of this study. Alternatively, they may have arisen through horizontal transfer of this bla_NDM-5_ into other *E. coli* sequence types.

The definitive cause of the category errors observed in our study among *E. coli* isolates is unknown; however, since all errors were observed across a wide range of MIC values (2/4–16/4 µg/mL), it seems unlikely that these errors could be attributed solely to a specific resistance phenotype. It is possible that the reduced performance of aztreonam–avibactam gradient strips may be observed in *E. coli* isolates; however, further testing is needed to definitively establish this limitation. Finally, since two out of three *E. coli* isolates that were not in category agreement were in essential agreement, these differences may represent expected assay variation near the interpretive breakpoint rather than performance issues inherent to the gradient strip diffusion testing method.

While our data demonstrate acceptable performance and feasible use of aztreonam–avibactam gradient strip diffusion testing, our study has several limitations. First, clinical isolates used in the study were obtained from a single geographical region and may not represent the breadth of MBL-producing Enterobacterales found globally. The inclusion of isolates from the CDC AR Bank served to introduce bacterial isolates from regions outside the New York City Metropolitan Area. An additional study limitation is the relatively small number of bacterial isolates exhibiting elevated aztreonam–avibactam MICs, which limits the ability of this study to assess very major errors. However, given the recent recommended use of this drug combination for treatment of infections with MBL-producing Enterobacterales*,* this limitation is expected. Finally, testing in this study was performed using culture media obtained from a single manufacturer. Prior studies describing broth disk elution for aztreonam–avibactam have noted differences in performance when culture media from different manufacturers is used (14). Additional studies may be required to determine if similar variations impact gradient strip diffusion methods.

Overall, our study demonstrates that determination of minimal inhibitory concentrations for aztreonam–avibactam using gradient strip diffusion is a feasible option for implementation in routine clinical laboratory testing procedures. This testing can be easily implemented as part of an antimicrobial reporting cascade for metallo-β-lactamase-producing Enterobacterales.

## SUPPLEMENTAL MATERIAL

Figure S1 jcm.00649-24-s0001.tif MIC distribution for all isolates in the study.

Supplemental legends jcm.00649-24-s0002.docx Legends for Table S1 and Figure S1.

Table S1 jcm.00649-24-s0003.docx CDC AR Bank isolates included in the study.

ASM does not own the copyrights to Supplemental Material that may be linked to, or accessed through, an article. The authors have granted ASM a non-exclusive, world-wide license to publish the Supplemental Material files. Please contact the corresponding author directly for reuse.
