# Supplementary figures and images for: Evaluation of gradient strip diffusion for susceptibility testing of aztreonam–avibactam in metallo-β-lactamase-producing Enterobacterales
Source: J Clin Microbiol. 2024 Sep 30;62(11):e00649-24. doi: 10.1128/jcm.00649-24 (PMC11559034; doi:10.1128/jcm.00649-24)

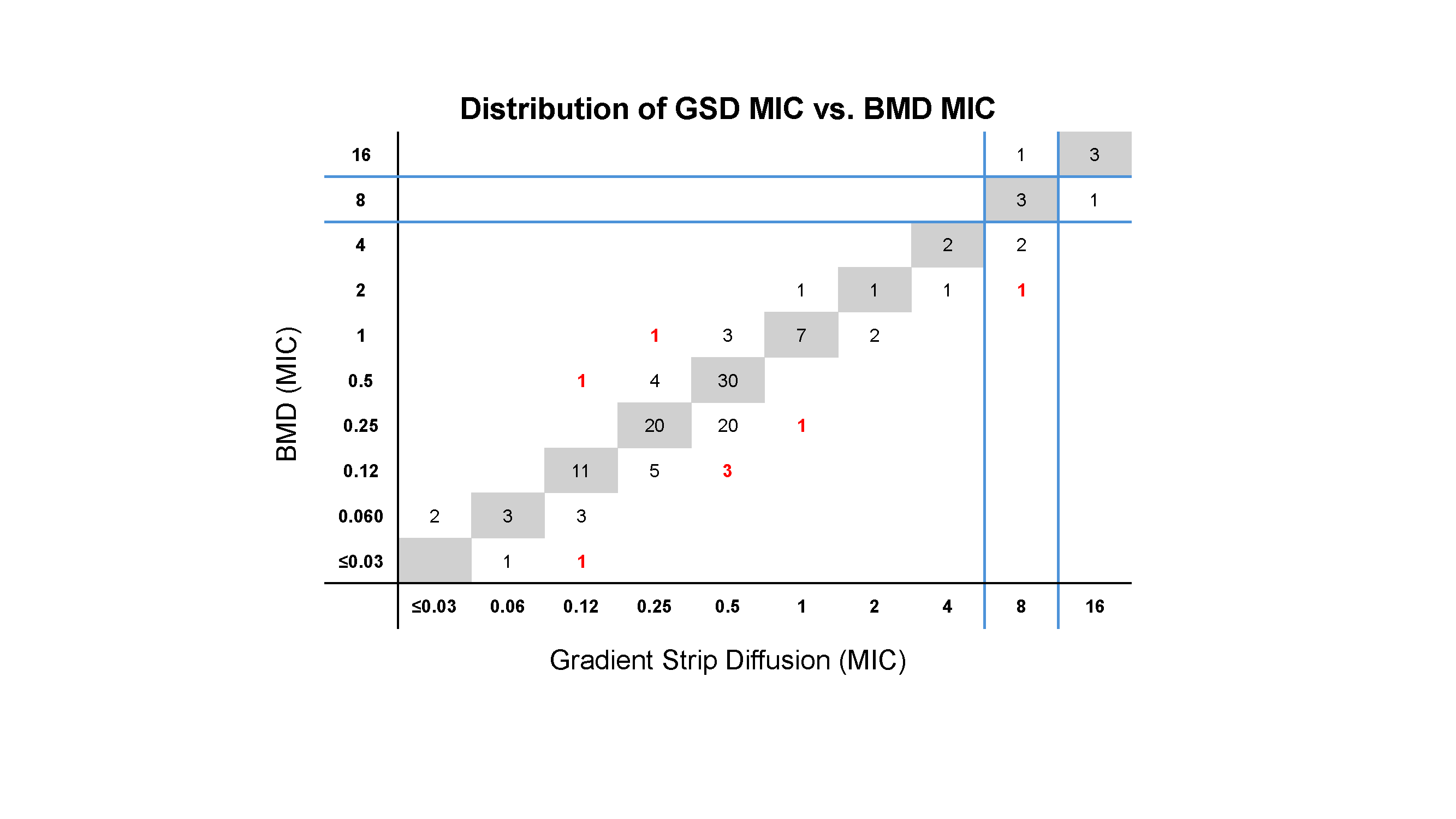

Supplement: Figure S1 — MIC distribution for all isolates in the study. [file jcm.00649-24-s0001.tif]
