# Supplementary material for: Evaluation of gradient strip diffusion for susceptibility testing of aztreonam–avibactam in metallo-β-lactamase-producing Enterobacterales
Source: J Clin Microbiol. 2024 Sep 30;62(11):e00649-24. doi: 10.1128/jcm.00649-24 (PMC11559034; doi:10.1128/jcm.00649-24)
Supplement: Supplemental legends — Legends for Table S1 and Figure S1. [file jcm.00649-24-s0002.docx]

Supplemental Table 1: List of CDC AR Bank Isolates Included in Study. This table shows the 31 CDC AR Bank isolates included in the evaluation of aztreonam-avibactam gradient strips. The table indicates the CDC AR Bank ID number, the organism identification, and the carbapenemase genes identified in each organism as annotated by the CDC AR Bank.

*Supplemental Figure 1: Distribution of Aztreonam-Avibactam Gradient Strip Diffusion MIC Compared to Broth Microdilution for Clinical Isolates and CDC AR Bank Isolates.* The distribution of aztreonam-avibactam MIC as tested by gradient strip diffusion (GSD) compared to the MIC measured by the comparator method (broth microdilution, BMD) for both the clinical (n = 103) and CDC AR Bank (n = 31) isolates included in this study is shown. Isolates with perfect MIC correlation are shaded in grey and majority of isolates (80/134, 59.7%) were in this category. Isolates with a GSD MIC that measured two or greater doubling-dilutions different from the comparator BMD MIC are highlighted in red and a minority of isolates (8/134, 6.0%) were in this category. There are currently no published interpretive breakpoints for aztreonam-avibactam. Blue lines represent the Clinical and Laboratory Standards Institute (CLSI) breakpoints for aztreonam as a surrogate for proposed aztreonam-avibactam breakpoints. Using these surrogate breakpoints, there were three isolates that would have incorrect interpretations by GSD. All three would be categorized as minor errors.
