# Supplementary material for: Evaluation of gradient strip diffusion for susceptibility testing of aztreonam–avibactam in metallo-β-lactamase-producing Enterobacterales
Source: J Clin Microbiol. 2024 Sep 30;62(11):e00649-24. doi: 10.1128/jcm.00649-24 (PMC11559034; doi:10.1128/jcm.00649-24)
Supplement: Table S1 — CDC AR Bank isolates included in the study. [file jcm.00649-24-s0003.docx]

| **AR Bank Number** | **Organism Name** | **Beta-Lactamase Genes** |
| --- | --- | --- |
| 2 | Enterobacter cloacae | KPC-3, ACT-45, OXA-9, TEM-1A |
| 3 | Klebsiella pneumoniae | KPC-3 |
| 6 | Escherichia coli | CMY-2 |
| 7 | Klebsiella aerogenes | Unknown |
| 21 | Citrobacter freundii | CMY-108 |
| 27 | Serratia marcescens | SME |
| 28 | Klebsiella oxytoca | OXY-1-1 |
| 34 | Klebsiella pneumoniae | IMP-4 |
| 38 | Enterobacter cloacae | NDM-1 |
| 40 | Klebsiella pneumoniae | VIM-27 |
| 48 | Escherichia coli | NDM-1, CMY-6 |
| 62 | Klebsiella aerogenes | CMY-2 |
| 68 | Klebsiella pneumoniae | NDM-1, OXA-232 |
| 69 | Escherichia coli | NDM-1 |
| 76 | Klebsiella pneumoniae | VIM-1 |
| 81 | Escherichia coli | CMY-2, TEM-1, ACRF |
| 97 | Klebsiella pneumoniae | KPC-3 |
| 129 | Klebsiella pneumoniae | KPC-3 |
| 135 | Klebsiella pneumoniae | VIM-1 |
| 137 | Escherichia coli | NDM-6, CMY-42 |
| 146 | Klebsiella pneumoniae | NDM-1 |
| 149 | Escherichia coli | NDM-7 |
| 151 | Escherichia coli | NDM-5, CMY-42 |
| 1149 | Klebsiella pneumoniae | IMP-18, KPC-45 |
| 1150 | Enterobacter cloacae complex | IMP-13, KPC-3 |
| 1151 | Enterobacter cloacae complex | NDM-1, KPC-3 |
| 1152 | Enterobacter cloacae complex | NDM-7, KPC-3 |
| 1153 | Klebsiella pneumoniae | NDM-1, OXA-181, KPC-2 |
| 1158 | Klebsiella pneumoniae | NDM-5, VIM-2, OXA-232 |
| 1160 | Enterobacter cloacae complex | ACT-89 |
| 1162 | Escherichia coli | NDM-5, OXA-48 |
